# Supplementary material for: Conditional chemoconnectomics (cCCTomics) as a strategy for efficient and conditional targeting of chemical transmission
Source: eLife. 2024 Apr 30;12:RP91927. doi: 10.7554/eLife.91927 (PMC11060718; doi:10.7554/eLife.91927)
Supplement: Figure 3—source data 3. [file elife-91927-fig3-data3.zip › Figure 3 source data3 part 2.zip!/Figure 3-Source data 3-part2/1060f2-1-CRISPResso_Report_gl5umwyq.zip!/CRISPResso_on_1060f2_2/9.Alleles_frequency_table_around_sgRNA_gcccacttcctggagtatcg.pdf]

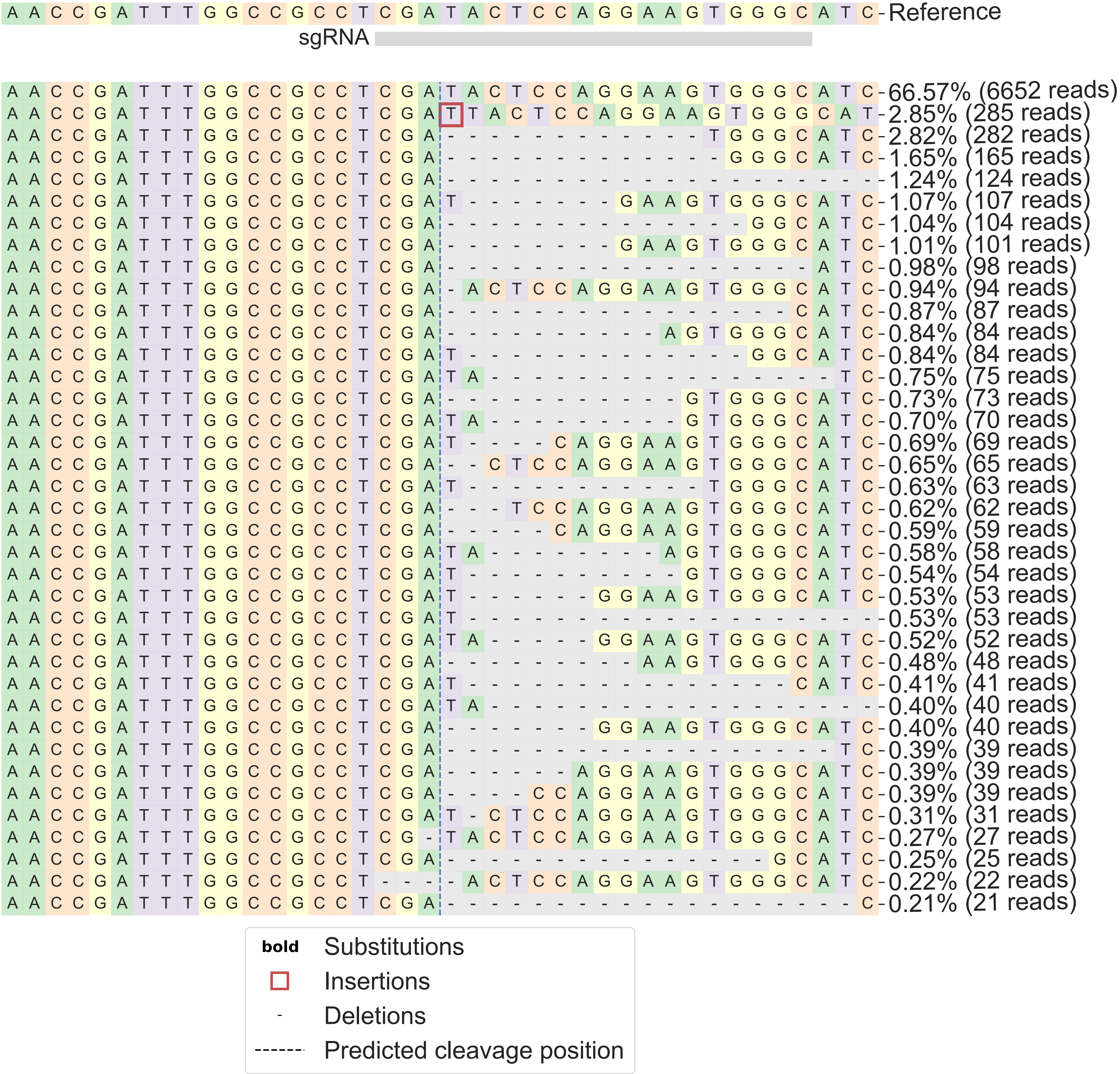
A A C C G A T T T G G C C G C C T C G A T A C T C C A G G A A G T G G G C A T C - Reference
sgRNA
66.57% (6652 reads)
2.85% (285 reads)
2.82% (282 reads)
1.65% (165 reads)
1.24% (124 reads)
1.07% (107 reads)
1.04% (104 reads)
1.01% (101 reads)
0.98% (98 reads)
0.94% (94 reads)
0.87% (87 reads)
0.84% (84 reads)
0.84% (84 reads)
0.75% (75 reads)
0.73% (73 reads)
0.70% (70 reads)
0.69% (69 reads)
0.65% (65 reads)
0.63% (63 reads)
0.62% (62 reads)
0.59% (59 reads)
0.58% (58 reads)
0.54% (54 reads)
0.53% (53 reads)
0.53% (53 reads)
0.52% (52 reads)
0.48% (48 reads)
0.41% (41 reads)
0.40% (40 reads)
0.40% (40 reads)
0.39% (39 reads)
0.39% (39 reads)
0.39% (39 reads)
0.31% (31 reads)
0.27% (27 reads)
0.25% (25 reads)
0.22% (22 reads)
0.21% (21 reads)
bold Substitutions
Insertions
Deletions
Predicted cleavage position
